# Does war increases the risk for psychoses?

**DOI:** 10.1192/j.eurpsy.2024.66

**Published:** 2024-08-27

**Authors:** P. Falkai

**Affiliations:** Psychiatry, University of Munich, Munich, Germany

## Abstract

**Abstract:**

The World Health Organization (WHO) has stated that in situations of armed conflict, “Around 10 percent of the people who experience traumatic events will have serious mental health problems, and another 10 percent will develop behavior that will hinder their ability to function effectively.” Problems include post-traumatic stress disorder, anxiety, depression, substance misuse, and possibly precipitation of psychosis. War has a catastrophic effect on the health and well being of nations. Studies have shown that conflict situations cause more mortality and disability than any major disease. Only through a greater understanding of conflicts and the myriad of mental health problems that arise from them, coherent and effective strategies for dealing with such problems can be developed.

**Disclosure of Interest:**

None Declared

